# Medical Monopolies and General Practitioners

**Published:** 1888-11-24

**Authors:** 


### The Hospital

A Weekly Institutional Journal of

**Science, Medicine, Nursing, and Philanthropy.**

*For Hospitals, Asylums, Medical Practitioners, Students, Nurses, Families, Parishes, Congregations, and the Charitable Public.*

Vol. V. No. 113.] [November 24, 1888.

### Medical Monopolies and General Practitioners.

"The price of liberty" is said to be "eternal vigilance." No less a price will secure for bodies of men or for individuals justice and fairplay. A rich old Yorkshireman who had once been poor used to say "the world is divided into two classes, the 'Haves' and 'Havenots.'" The "Haves" are always trying to keep what they have got, and the "Havenots" are always assailing their monopoly. A newspaper writer occupies a peculiar position with regard to the world's great cricket match. It is his duty to act as far as possible as an umpire, and to pronounce an impartial verdict between the "ins" and the "outs."

The medical profession, although but a small portion of the universal kosmos, is a world in itself. The public have the impression that it is a huge society of "wranglers," not in the mathematical, but in the "bread and butter" sense. Undoubtedly "doctors disagree." If we were to say otherwise, at any rate at the present moment, every European newspaper published during the last three months would give us the "lie direct." But doctors are not really worse than other people. There is a limited quantity of bread and butter available for distribution among them, and some of them insist upon having more than their share. The result is that others get too little, and not unnaturally complain of the fact.

These are the circumstances of the whole profession. There is a more limited area in which "wranglers" of another kind are the order of the day. Most people know that the chief object of a doctor's ambition is a hospital appointment. By preference it should be at a large general hospital, and, if possible, in connexion with a medical school. But if that may not be compassed, then a small general, special or Cottage hospital is not to be despised. Rather than a man of ill-regulated ambition will be without an appointment at all he will even choose to be junior assistant surgeon to the hospital for the mechanical elongation of abbreviated men, or pathologist to the asylum for the care and cure of impecunious husbands or of fashion-loving wives.

But, like the bread and butter spoken of before, hospital appointments are strictly limited in number, and that fact gives them their chief value. They constitute a narrow platform to which a very few may scramble up, and when they are once up they congratulate themselves and each other, whilst shaking a derisive fist at those below. Looked at impartially and without that heat which personal disappointment is apt to generate, there is nothing very uncommon or very surprising in all this. All doctors cannot be hospital physicians and surgeons, though some of them must be. The question is, "Which?" And here, perhaps, we come upon the very centre and prime secret of medical misunderstandings.

In choosing men for hospital appointments, it is obvious that some principle of selection must be adopted. It ought to be equally obvious that there is only one really sound principle, and that is to put the best and fittest men in. A large portion of the medical profession roundly affirm that this is one of the very last principles which any hospital committee ever thinks of adopting. A controversy has recently been carried on in the *British Medical Journal* which throws a good deal of light on this burning question. One correspondent points out that the "power of hanging on" has often a good deal to do with securing and retaining hospital appointments. What in the world can be meant by the "power of hanging on"? "Hanging on" to what? It really means the power of "hanging on" to professional idleness at home. "Hanging on" to a respectable house, and a neat man-servant, with three fairly sufficient meals a-day for ten or twelve years, until private patients find out the distinguished merits of the "hanger on." In short it means money. A man may ask and obtain a hospital appointment, suggests this correspondent, not on the strength of his merit, but on the length of his purse. If his father has been very successful in business or otherwise, and has left his son a modest fifteen or twenty thousand pounds, the young man can set up in practice as a "consultant," and despise his old college friends, whose want of the twenty thousand reduces them to the less distinguished level of industrious general practitioners.

Another correspondent uses the ill-omened word "boycotting" in connexion with hospital appointments. He says in effect that two or three favoured diplomas alone qualify for them. Those degrees are in no sense superior to a dozen other qualifications which might be named. They are, in fact, qualifications which certain colleges of physicians and surgeons alone have the power to give. The University of London, the Universities of Oxford, Cambridge, Edinburgh, and Dublin all confer degrees of the very highest class. Yet not one of these is by itself sufficient to qualify the most distinguished graduate of his year for the post of physician to the Mudlington Surburban Hospital for diseases of the lobe of the right ear. It is to be noticed that none of the correspondents blame the colleges. The colleges, indeed, are not at fault; but the hospitals. And yet it is not the hospitals, but the members of the medical and surgical staff of each institution. That is really where the whole difficulty lies. When a hospital has a vacant appointment for a physician or surgeon, how is it filled up? Apparently in the most open way, by publicly advertising in the medical newspapers and inviting candidates to apply. But what happens usually when replies to the advertisements are sent in? All such replies are first of all submitted to the medical committee. They employ their winnowing fans upon them, and send up for the choice of the general committee perhaps three, perhaps two, or in some cases it may be only one name out of fifty or a hundred. What principle of selection have they adopted? They have first applied the ridiculous and antiquated bye-law which still has place in the rules of almost all hospitals, and which provides that all candidates must be members or fellows of one, two, or three particular colleges. By this means they often succeed in disposing of four-fifths of their candidates, and not seldom they thereby exclude all the best men. That particular bye-law would be altered at every hospital in the kingdom within twelve months if it were not for the unstatesmanlike mental density of the honorary medical officers of the institutions.

Two particular consequences, among many others, are said to arise from this purely trades-union and irrational regulation. Some correspondents affirm that in many instances only the second or third best men become hospital physicians, and that as a result the patients are not treated with the highest available skill, whilst the medical profession is retarded in its forward advance. That is one series of consequences. Another result is that the bulk of the profession pass their lives without hope of promotion or stimulus to scientific exertion. One gentleman declares that once a man is fully committed to general practice he is "done for." He has entered an "Inferno" over the portals of which may be inscribed the dread words which Dante and Virgil saw over the entrance to another Inferno—"All hope abandon ye who enter here." No amount of professional skill or personal merit can ever secure for a general practitioner, whilst he remains a general practitioner, that recognition and distinction which are his due. If this be so, and it undoubtedly is, hospitals are inflicting grievous injury on a great profession.

Whilst we admit the services hospitals render to young men in the way of education, we cannot deny that those services may be more than counterbalanced by the dead weight of their foolish regulations, which condemn ninety-nine in every hundred medical men to the hopeless level of private soldiers during the whole of their professional life. If all hospitals would make a regulation that no man under forty years of age should be appointed full physician, and no man under thirty-five full surgeon, except in cases of absolute necessity, and that with these limitations all hospital appointments should be open to the whole profession, there would be reason in it. They would then have for their medical officers proved and experienced men, and not as they now often have, mere academic and unpractical "hangers on." It is high time that the whole question of hospital and other public appointments was reconsidered from the standpoint of public well-being and professional progress. Monopolies are generally detestable, but they surely reach a maximum of detestability when they are carried on at the expense of patients' lives and the retardation of the progress of scientific medicine.

